# Unraveling the effects of glycerophosphoinositol-enriched hyaluronic acid eyedrops on the ocular surface in glaucoma: a report from the multicenter OSGLA registry

**DOI:** 10.3389/fmed.2026.1868619

**Published:** 2026-08-06

**Authors:** Luca Agnifili, Davide Celani, Maria Ludovica Ruggeri, Alessandro Sferra, Rodolfo Mastropasqua, Francesco Oddone, Carmela Carnevale, Antonio Pinna, Michele Figus, Matteo Sacchi

**Affiliations:** 1 Ophthalmology Clinic, Department of Medicine and Ageing Science, University “G. d'Annunzio” of Chieti-Pescara, Chieti, Italy; 2 Alma Mater Studiorum, University of Bologna, Bologna, Italy; 3 Ophthalmology Clinic, SS Annunziata Hospital, Chieti, Italy; 4 Ophthalmology Clinic, Department of Neuroscience, Imaging and Clinical Science, “G. d'Annunzio” University Chieti-Pescara, Chieti, Italy; 5 IRCCS Fondazione Bietti, Rome, Italy; 6 Department of Medicine, Surgery and Pharmacy, University of Sassari, Sassari, Italy; 7 Ophthalmology Unit, Department of Surgery, Medicine, Molecular and Emergency, University of Pisa, Pisa, Italy

**Keywords:** dry eye, glaucoma, glaucoma therapy-related ocular surface disease, glycerophosphoinositol, hyaluronic acid

## Abstract

**Purpose:**

To evaluate the effects of 0. 001% glycerophosphoinositol in 0.2% hyaluronate (GPI/HA) vehicle eye drops in patients with glaucoma therapy related-ocular surface disease (GTOSD).

**Methods:**

Forty patients diagnosed with GTOSD and receiving GPI/HA or HA 0.2%, underwent ocular surface disease index (OSDI), Symptom Assessment iN Dry Eye (SANDE), National Eye Institute Visual Function Questionnaire (NEI VFQ)-25 questionnaires, and tear matrix metalloproteinase-9 (MMP-9), break-up time (BUT), corneal fluorescein staining (CFS), Schirmer test I (STI), and bulbar conjunctival hyperemia (BCH) determination

**Results:**

After 1 month, GPI/HA eyedrops increased BUT [6 (4–7.25) to 7 (6–8)] and STI [11.5 (8–17.5) to 13.5 (10–18.5)], whereas decreased BCH [2.2 (2.1–2.3) to 2.1 (1.3–2.2)] and CFS [3 (2–4) to 2 (2–3)] (*p* < 0.001). Questionnaire scores improved from 35 (24.36–46.25) to 33 (20.75–40.52), for SANDE and from 31.5 (20–45) to 28.5 (17.75–40.5), for OSDI (*p* < 0.001). HA 0.2% eyedrops reduced BCH from 2.75 (2.15–3.20) to 2.25 (1.90–2.30) and CFS from 3.5 (2.75–5) to 2.5 (2–4) (*p* = 0.014), and increased BUT (*p* = 0.036). Questionnaire scores improved from 28.95 (20.9–47.6) to 26.86 (19.90–36.41) (*p* = 0.009) for SANDE, and from 40 (19.5–49) to 29 (19.75–42) (*p* = 0.005) for OSDI. There were not significant modifications in the MMP-9 immunoassay positivity in either treatment group. Follow-up inter-group comparisons showed better BCH values in GPI/HA, and better NEI VFQ-25 scores in HA 0.2%.

**Conclusion:**

Both treatments proved valuable in managing GTOSD, supporting the use of lubricants in this condition. GPI/HA showed a broader pattern of improvement, which should be interpreted cautiously given the observational design and limited inter-group differences.

## Introduction

Glaucoma therapy-related ocular surface disease (GTOSD) is a frequent and clinically meaningful comorbidity in patients receiving long-term topical intraocular pressure (IOP) lowering therapy. Chronic treatment induces a plethora of ocular surface alterations—including epithelial damage, tear film instability, goblet cell loss, meibomian gland dysfunction, conjunctival inflammation, and immune activation—that resembles an iatrogenic form of dry eye disease (1, 2). These changes arise from the pharmacological effects of active compounds and from preservatives, which promote toxicity, oxidative stress, para-inflammatory activation, upregulation of inflammatory mediators such as matrix metalloproteinases (MMP)-9 (3–5). GTOSD significantly affects glaucoma management, being associated with reduced treatment adherence, poorer quality of life and disease control, and unfavorable surgical outcomes (6–8).

Despite its clinical relevance, evidence-based management strategies to mitigate GTOSD remain limited, and most supportive interventions—including therapy simplification, the use of preservative-free IOP lowering drugs, and tear substitutes—are applied with variable success (6, 7).

Hyaluronic acid (HA) is widely used for its lubricating and epithelia-protective properties, while increasing attention has been directed toward formulations enriched with alternative, non-pharmacological anti-inflammatory molecules, such as glycerophosphoinositol (GPI), a naturally occurring phosphatidylinositol derivative with non-steroidal immunomodulatory activity (9, 10).

Experimental studies demonstrated its ability to downregulate pro-inflammatory pathways, reduce cytokine-mediated epithelial stress, and modulate MMP activity with an efficacy profile comparable to corticosteroids, but without their limitations (11). Recent clinical findings reported in non-glaucomatous populations, such as patients with pollutants triggered allergic conjunctivitis (the urban syndrome), suggest that topical GPI in HA vehicles improves symptoms and signs of OSD, supporting its potential role as a safe and well-tolerated adjunctive therapy in chronic, low-grade inflammatory conditions (10, 12).

To date, the effects of GPI-enriched eyedrops in glaucomatous patients with GTOSD remain unexplored. The Ocular Surface & Glaucoma (OSGLA) Registry is a structured, multicenter real-world platform specifically designed to characterize GTOSD in patients receiving medical glaucoma therapy (5).

Here we report an observational sub-analysis of the OSGLA Registry aimed at evaluating the effects of a preservative-free GPI/high-molecular-weight hyaluronic acid formulation on ocular surface signs, symptoms, and tear inflammatory markers.

## Materials and methods

### OSGLA registry

The Ocular Surface & Glaucoma (OSGLA) Registry is an ongoing prospective, multicenter observational initiative designed to collect standardized real-world data on ocular surface alterations in patients with glaucoma receiving medical therapy. The registry systematically collects clinical, functional, and patient-reported outcomes to characterize the spectrum of glaucoma therapy-related ocular surface disease (GTOSD), following a unified data acquisition protocol across participating centers. Therapeutic management remains at the discretion of the treating physician. All procedures adhere to the Declaration of Helsinki, and written informed consent was obtained from all participants. The registry was approved by the Institutional Review Board of the coordinating center (Department of Medicine and Aging Science, University “G. d'Annunzio” of Chieti-Pescara, approval no. 2024.0121).

The primary outcomes of the OSGLA Registry are measurements of longitudinal changes in the GTOSD severity, assessed by the ocular surface disease index (OSDI) score, tear film break-up time (BUT), Schirmer's test I (STI), corneal fluoresceine staining (CFS), bulbar conjunctiva hyperemia (BCH), and tear matrix metalloproteinase MMP-9 immunoassay positivity. Secondary outcomes include Symptom Assessment iN Dry Eye (SANDE) and National Eye Institute Visual Function Questionnaire (NEI VFQ)-25 scores, IOP, visual field and optic nerve parameters, adherence to therapy information, treatment switches, and surgical events.

To ensure consistency across centers, trained investigators follow a harmonized evaluation protocol.

Eligible patients are adults diagnosed with primary open-angle glaucoma or ocular hypertension who have been on stable topical therapy for at least 6 months. Glaucoma severity was classified according to the Hodapp–Parrish–Anderson criteria based on visual field mean deviation (MD), with early, moderate, and advanced disease defined as MD ≥ −6 dB, MD < −6 to ≥ −12 dB, and MD < −12 dB, respectively (13).

GTOSD is identified based on an OSDI score above 13 combined with at least one abnormal ocular surface parameter such as reduced BUT, presence of CFS, or increased BCH. Patients are excluded if they have undergone recent ocular surgery, present ocular comorbidities unrelated to glaucoma or GTOSD, use topical or systemic treatments that may interfere with tear film function, or have systemic inflammatory conditions known to affect the ocular surface. Patients who underwent phacoemulsification are allowed to be included after a minimum period of at least 6 months. Contact lens wear, active infections, pregnancy, breastfeeding, and instillation of topical lubricants during the month preceding enrollment also constituted exclusion criteria.

Registry visits include baseline evaluation, a 1-month follow-up, visits following therapeutic modifications, and routine monitoring approximately every 4 months. All assessments are performed by the same investigator at each center to minimize inter-observer variability.

### Study cohort and treatments

This retrospective sub-analysis of the OSGLA Registry compared glaucoma patients receiving adjunctive treatment with a preservative-free formulation containing 0.001% glycerophosphoinositol in 0.2% high-molecular-weight hyaluronic acid (GPI/HA) (Corneial^®^ eye drops, Biofta, Bionativa S.p.A., Barberino Tavarnelle, FI, Italy) with patients receiving preservative-free 0.2% high-molecular-weight hyaluronic acid alone (HA) (Wiky eye drops, Esoform S.p.A., Rovigo, Italy).

All 40 Registry patients who met the eligibility criteria for the present sub-analysis and had complete baseline and 1-month follow-up data were included. Of these, 28 received adjunctive GPI/HA therapy and 12 received HA 0.2% alone. Supportive therapy was prescribed as part of routine clinical practice to manage GTOSD, with treatment selection made by the treating physician and without random allocation.

Eligible patients had a diagnosis of open-angle glaucoma or ocular hypertension, were on stable topical hypotensive therapy, and showed clinical evidence of GTOSD with complete baseline and follow-up data for primary outcomes. Patients with ocular or systemic conditions affecting tear film homeostasis or recent topical anti-inflammatory treatment were excluded. Baseline IOP-lowering treatment characteristics, including the number and classes of active compounds, number of daily instillations, use of preservative-free medications, and exposure to BAK-preserved medications, were recorded and compared between treatment groups. No changes in IOP-lowering therapy occurred between baseline and the 1-month follow-up.

### Clinical examination protocol

All evaluations followed the standardized OSGLA procedures. Ocular surface symptoms were assessed using the OSDI and a visual analog scale that explored the intensity of discomfort and dryness. Tear film stability was evaluated through fluorescein tear BUT, measured after instillation of fluorescein dye and using the average of three consecutive measurements. Tear production was assessed with the STI, performed without anesthesia, and read after 5 min. BCH was graded via slit-lamp examination using a standard four-step severity scale (14). CFS was evaluated according to the Oxford grading scheme, considering punctate staining in all five corneal regions (15).

Ocular surface examinations were performed in the order suggested by the DEWS guidelines (16). Tear film inflammation was explored through the assessment of MMP-9 using a commercially available point-of-care immunoassay, with results reported as positive or negative according to manufacturer thresholds.

### Outcomes of the study

The main clinical outcomes of interest were changes from baseline to 1 month in BCH, CFS, BUT, and STI. Patient-reported questionnaire scores were additional patient-reported outcomes, while changes in MMP-9 immunoassay positivity were analyzed exploratorily.

The primary aim of the present study was to assess changes in clinical ocular surface parameters 1 month following adjunctive treatment with GPI/HA or HA. Questionnaire scores were considered secondary outcomes, whereas changes in tear MMP-9 immunoassay positivity and its correlations with clinical ocular surface parameters and questionnaire scores were evaluated as exploratory outcomes.

### Statistical analysis

Statistical analyses were performed using the R software environment for statistical computing and graphics (version 4.4.3; R Foundation for Statistical Computing, Vienna, Austria). Data normality was assessed using the Kolmogorov–Smirnov, Shapiro-Wilk, and Jarque-Bera tests, along with visual inspection of Q–Q plots. As most variables showed non-normal distributions, non-parametric statistical methods were applied. Descriptive statistics were therefore reported as median and interquartile range (IQR) [first and third quartiles].

Pre- and post-treatment comparisons were conducted using the Wilcoxon signed-rank test. Intergroup comparisons at baseline and follow-up were performed using the Mann-Whitney *U* test. To quantify the magnitude of within-group changes and between-group differences, non-parametric effect sizes (*r*) were calculated based on the corresponding Wilcoxon signed-rank and Mann–Whitney U tests. Effect sizes were interpreted according to commonly adopted thresholds for non-parametric effect size *r*, with values of approximately 0.10, 0.30, and ≥ 0.50 considered indicative of small, moderate, and large effects, respectively. No parametric effect size measures were applied, as the data distribution did not meet parametric assumptions.

Statistically significant differences were illustrated using boxplots.

For the binary variable MMP-9 levels (positive vs. negative), within-group comparisons between baseline and follow-up were performed using the McNemar test. Correlations between MMP-9 immunoassay results and clinical parameters were evaluated using Spearman's rank correlation coefficient, given the non-normal distribution of the variables. Given the exploratory nature of the study and the absence of an *a priori* sample size calculation, treatment-associated changes were assessed through non-parametric effect sizes (*r*), rather than *post-hoc* power calculations, which may provide misleading estimates when applied retrospectively to exploratory studies. All statistical tests were two-sided, and a *p*-value ≤ 0.05 was considered statistically significant.

## Results

A total of 40 patients (40 eyes, one eye per patient) were included in the study, of whom 28 received adjunctive GPI/HA therapy and 12 received HA 0.2% alone. Since only one eye per patient was included, each patient contributed a single ocular observation, and all statistical analyses were performed at the patient level. All patients completed the scheduled 1-month follow-up assessment performed after treatment initiation, with no loss to follow-up and no missing data recorded. Therefore, no imputation methods were required. Baseline demographic and clinical characteristics are reported in Table 1.

**Table 1:** Baseline and follow-up demographic and clinical features.

Treatment regimen	Gender (M/F)	Age (years)	Baseline IOP (mmHg)	1-month IOP (mmHg)	MD (db)	Duration of IOP lowering therapy (y)	N. of IOP lowering medications	BCVA	Status of the lens (P/Ps)
GPI/HA	15/13	71 (62.75–76.25)	18.5 (16–21)	18.3 (16–21)	−3.6 (−6.45/−2.74)	10 (5–13.5)	1 (1–2)	3.5 (1–5)	15/13
HA	8/4	67 (58.5–71.5)	18.5 (15.75–20.25)	18.3 (15.50–20.50)	−3.16 (−4.37/−2.09)	9 (5.75–13.5)	1 (1–2)	1 (1–1.5)	7/5

GPI, glycerophosphoinositol; HA, hyaluronic acid; IOP, intraocular pressure; M/F, male/female; MD, mean deviation; BCVA, best corrected visual acuity; P/Ps, phakic/pseudophakic. *p* > 0.05 for all between-group comparisons.

*Data are expressed as median and interquartile range (Q1*–*Q3)*.

Baseline characteristics of IOP-lowering therapy and preservative exposure are reported in Table 2. No significant differences between groups were observed in the number of active compounds, pharmacological classes, number of daily instillations, use of preservative-free medications, or exposure to BAK-preserved medications (all *p* > 0.05).

**Table 2:** Baseline characteristics of IOP-lowering therapy and preservative exposure in the two treatment groups.

Baseline glaucoma therapy	HA (*n* = 12)	GPI/HA (*n* = 28)	*p*-value
Number of active compounds, median (IQR)	2 (1.5–2.5)	2 (1.5–2.5)	0.171
PGA, *n* (%)	9 (75%)	23 (82%)	0.211
BB, *n* (%)	5 (42%)	13 (46%)	0.230
AA, *n* (%)	2 (17%)	6 (21%)	0.318
CAI, *n* (%)	4 (33%)	8 (29%)	0.255
Number of instillations per day, median (IQR)	1.5 (1.0–2.0)	1.5 (1.0–2.0)	0.341
PF medications (≥1), *n* (%)	6 (50%)	16 (57%)	0.199
BAK-preserved medications (≥1), *n* (%)	8 (66%)	17 (61%)	0.263

Continuous variables are expressed as median and interquartile range (IQR; Q1–Q3), and categorical variables as *n* (%).

No exposure to preservative-containing formulations other than BAK-preserved medications was recorded.

*GPI, glycerophosphoinositol; HA, hyaluronic acid; IOP PGA, prostaglandin analog; BB, beta blocker; AA, alpha agonist; CAI, carbonic anhydrase inhibitor; PF, preservative-free; BAK, benzalkonium chloride*.

There were no significant baseline differences between groups, except for BCH, which was significantly lower in the GPI/HA group. Overall, follow-up outcomes revealed a statistically significant improvement in most ocular surface parameters and questionnaire scores in both treatment regimens (Table 3). No topical or systemic adverse events were reported in either treatment regimen.

**Table 3:** Baseline and follow-up ocular surface clinical tests, questionnaire scores, and MMP-9 immunoassay positivity.

Variable	HA			GPI/HA		
	Baseline	Follow-up	*p*-value	Baseline	Follow-up	*p*-value
BCH *score*	2.75 (2.15–3.20)	2.25 (1.90–2.30)	*0.014*	2.2 ^*^ (2.1–2.3)	2.1 (1.3–2.2)^†^	* < 0.001*
CFS *score*	3.5 (2.75–5)	2.5 (2–4)	*0.014*	3 (2–4 m)	2 (2–3)	* < 0.001*
BUT *(sec)*	6 (5–6.25)	6 (5.75–8)	*0.036*	6 (4–7.25)	7 (6–8)	* < 0.001*
STI *(mm)*	11.5 (4.75–18.25)	14 (7.75–16.25)	*0.365*	11.5 (8–17.5)	13.5 (10–18.5)	*0.009*
SANDE score	28.95 (20.9–47.6)	26.86 (19.90–36.41)	*0.009*	35 (24.36–46.25)	33 (20.75–40.52)	* < 0.001*
OSDI *score*	40 (19.5–49)	29 (19.75–42)	*0.005*	31.5 (20–45)	28.50 (17.75–40.5)	* < 0.001*
NEI VFQ-25 *score*	69.05 (62.67–76.25)	77.22 (65.40–78.85)	*0.064*	58.1 (47.25–70)	63.19 (50.25–70.33)^‡^	*0.395*
MMP-9 immunoassay	4/8	6/6	*0.479*	11/17	14/14	*0.248*

HA, hyaluronic acid; GPI, glycerophosphoinositol, BCH: bulbar conjunctiva hyperemia, CFS, corneal fluorescein staining; BUT, break-up time; STI, Schirmer test I; SANDE, Symptoms Assessment iN Dry Eye; OSDI, ocular surface disease index; NEI VFQ-25, National Eye Institute Visual Function Questionnaire; MMP, matrix metalloproteinase.

^*^p = 0.034 vs baseline HA 0.2%;^†^*p* = 0.047 vs. follow-up HA 0.2%; ^‡^*p* = 0.014 vs. follow-up HA 0.2%.

*Data are expressed as median and interquartile range (Q1-Q3)*.

### HA treatment regimen

**Figure 1:**
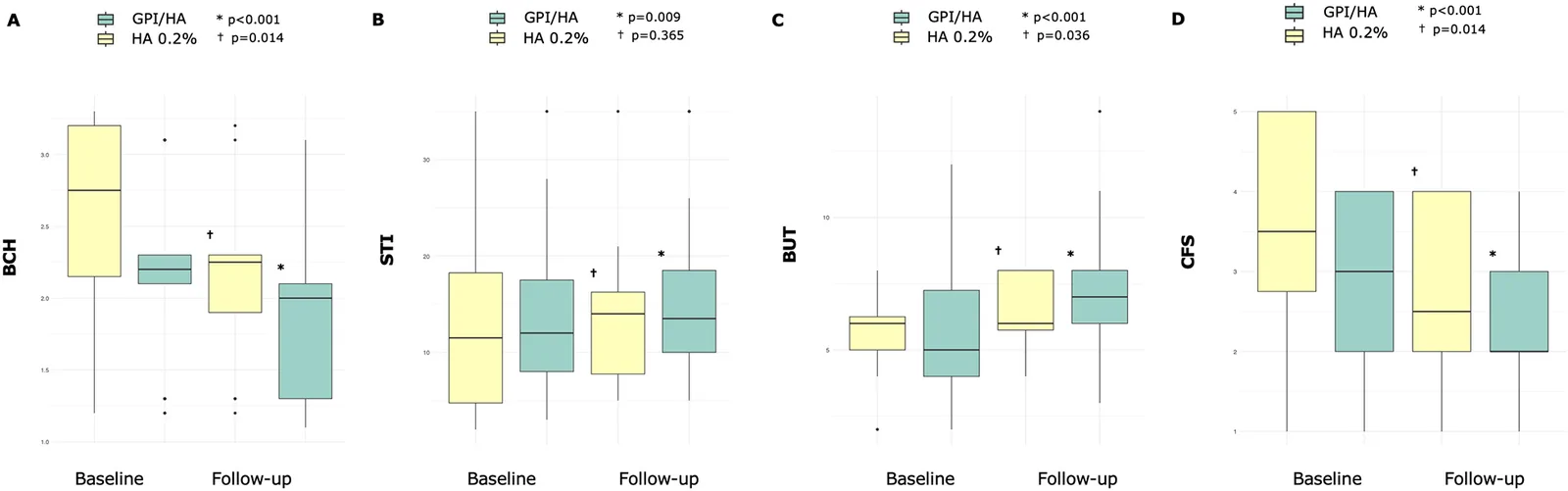
Baseline and 1-month median values of BCH **(A)**, STI **(B)**, BUT **(C)**, and CFS **(D)** in the GPI/ HA and HA treatment groups. Variables improved in both treatments, except for STI, with numerically greater improvements observed in patients treated with GPI/HA. GPI, glycerophosphoinositol; HA, hyaluronic acid; BCH, bulbar conjunctiva hyperemia; STI, Schirmer test I; BUT, break-up time; CFS, corneal fluoresceine staining.

In this treatment regimen, BCH significantly decreased by approximately 20%, from a median baseline value of 2.75 (IQR 2.15–3.20) to 2.25 (IQR 1.90–2.30) at follow-up (*p* = 0.014). Similarly, CFS score showed a significant reduction of about 30%, decreasing from 3.5 (IQR 2.75–5) to 2.5 (IQR 2–4) (*p* = 0.014), and median BUT significantly improved (*p* = 0.036). Conversely, no significant changes were detected in STI values (*p* = 0.365; Figure 1).

**Figure 2:**
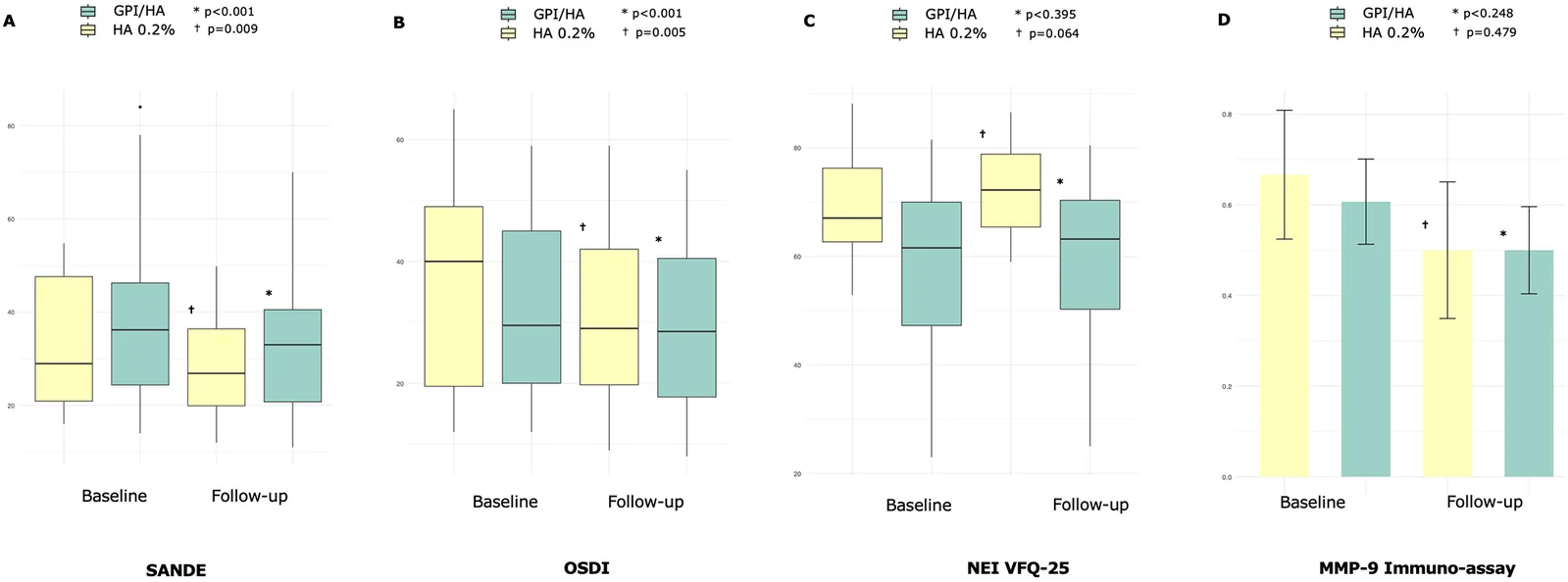
Baseline and one-month SANDE **(A)**, OSDI **(B)**, NEI VFQ-25 **(C)** and MMP-9 immuno-assay **(D)** scores in GPI/ HA and HA treatment groups. SANDE and OSDI scores improved in both treatments, with numerically greater improvements observed in patients treated with GPI/HA. GPI, glycerophosphoinositol; HA, hyaluronic acidl SANDE, Symptoms Assessment iN Dry Eye; OSDI, ocular surface disease index; NEI VFQ-25, National Eye Institute Visual Function Questionnaire-25.

**Figure 3:**
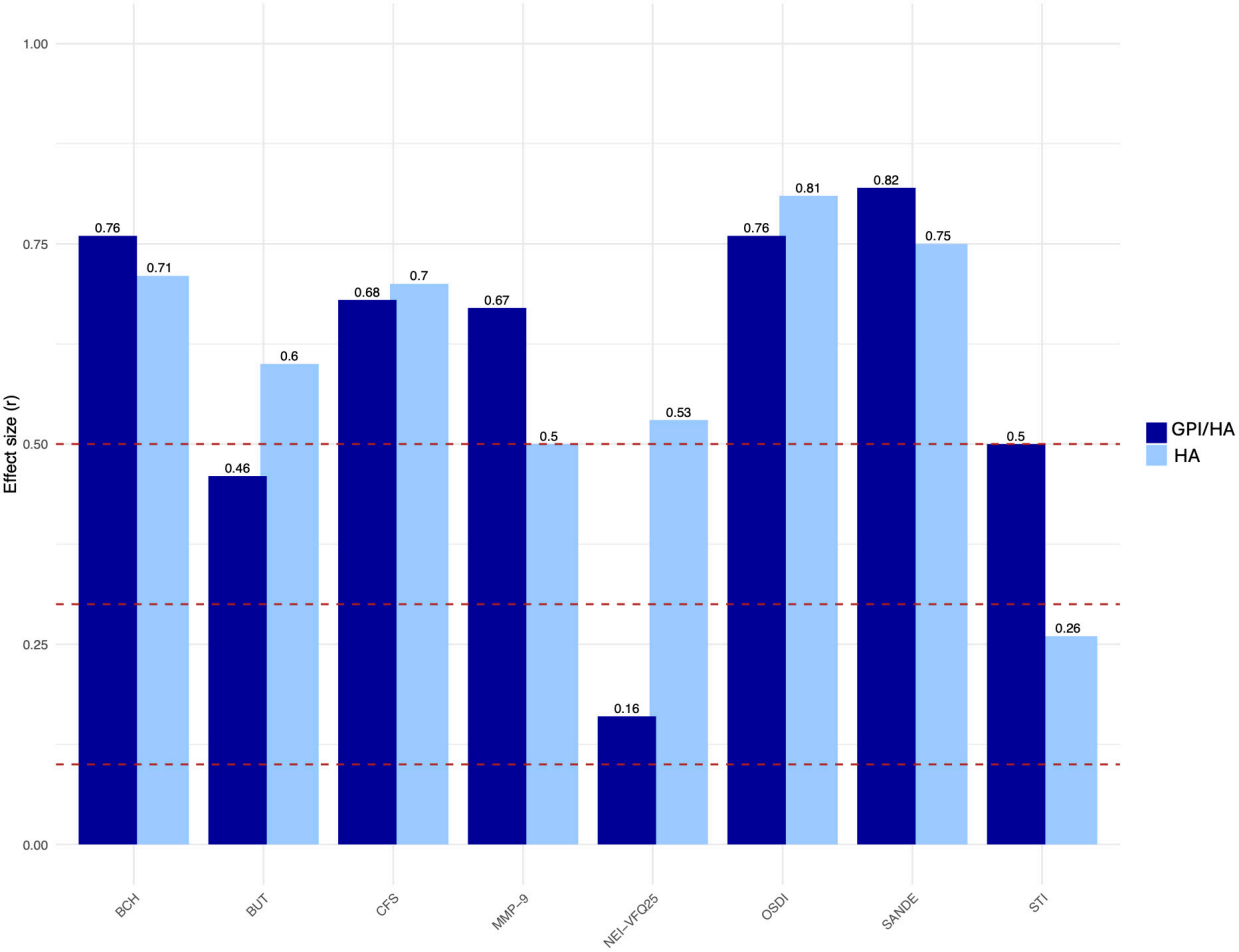
Effect sizes (r) of within-group changes in ocular surface clinical parameters and questionnaire scores after 1 month of adjunctive treatment with GPI/HA or HA 0.2% alone. Dashed lines indicate conventional thresholds for small (*r* = 0.1), moderate (*r* = 0.3), and large (*r* = 0.5) effect sizes. GPI, glycerophosphoinositol; HA, hyaluronic acid.

Regarding self-reported questionnaires, SANDE score significantly decreased by approximately 8%, from 28.95 (IQR 20.9–47.6) to 26.86 (IQR 19.90–36.41) (*p* = 0.009), while OSDI score declined by nearly 30%, from 40 (IQR 19.5–49) to 29 (IQR 19.75–42) (*p* = 0.005). Conversely, NEI VFQ-25 score and MMP-9 immunoassay positivity did not show significant changes (*p* = 0.064 and 0.479, respectively; Figure 2). Overall, we observed improvement in five parameters out of 8. When evaluating the magnitude of changes, large effect sizes were observed for OSDI (*r* = 0.81), SANDE (*r* = 0.75), BCH (*r* = 0.71), CFS (*r* = 0.70), and BUT (*r* = 0.60), respectively. The effect for STI (*r* = 0.26) was small (Figure 3).

### GPI/HA treatment regimen

Patients treated with GPI/HA showed improvements of several objective and subjective parameters. BCH score significantly decreased from 2.2 (IQR 2.1–2.3) at baseline to 2.1 (IQR 1.3–2.2) at follow-up (*p* < 0.001). Tear film stability improved by a 15%, with BUT values increasing from 6 (IQR 4–7.25) to 7 (IQR 6–8) s (*p* < 0.001). Tear volume, as assessed by STI, also showed an increase of 15%, with values improving from 11.5 (IQR 8–17.5) to 13.5 (IQR 10–18.5) mm (*p* = 0.009), while CFS score improved by one third, decreasing from 3 (IQR 2–4) to 2 (IQR 2–3) (*p* < 0.001; Figure 1). There were improvements of self-reported questionnaires, with SANDE score decreasing from 35 (IQR 24.36–46.25) to 33 (IQR 20.75–40.52) (*p* < 0.001) and OSDI showing a 10% reduction from 31.5 (20–45) to 28.5 (17.75–40.5) (*p* < 0.001). As for HA group, NEI VFQ-25 score and MMP-9 immunoassay positivity did not show significant changes (*p* = 0.395 and 0.248, respectively; Figure 2). Overall, we observed improvement in six parameters out of 8.

When evaluating the magnitude of changes in this group, large effect sizes were observed for SANDE (*r* = 0.82), OSDI (*r* = 0.76), BCH (*r* = 0.76), and CFS (*r* = 0.68). STI showed a moderate effect size (*r* = 0.50), while BUT demonstrated a moderate effect (*r* = 0.46; Figure 3).

### Intergroup comparisons

At baseline, BCH was significantly lower in GPI/HA treatment (*p* = 0.034; Table 3). No significant intergroup differences were found for BUT (*p* = 0.937), STI (*p* = 0.615), CFS (*p* = 0.162), OSDI (*p* = 0.647), SANDE (*p* = 0.813), and NEI VFQ-25 scores (*p* = 0.074).

At the 1 month follow-up, BCH values were significant lower in the GPI/HA treatment (*p* = 0.047) while NEI VFQ-25 score was significantly higher in the HA group (*p* = 0.014). For other parameters, such as BUT (*p* = 0.509), STI (*p* = 0.485), CFS (*p* = 0.336), OSDI (*p* = 0.883), and SANDE (*p* = 0.669) there were not significant differences between groups.

### Correlation analysis

In the HA group, MMP-9 immunoassay positivity showed positive correlations with BCH (ρ = 0.67) and CFS (ρ = 0.64), while a moderate negative correlation was found with BUT (ρ = −0.35). A strong and negative correlation was conversely found between MMP-9 immunoassay positivity and NEI VFQ-25 scores (ρ = −0.72). In the GPI/ HA group, correlations between MMP-9 immunoassay results and objective and subjective ocular surface parameters were weak. Only minimal associations were detected with BCH, CFS, BUT, STI, OSDI, SANDE, and NEI VFQ-25 scores (all |ρ| < 0.30), and all corresponding *p*-values were largely non-significant, with no trend toward statistical significance.

## Discussion

This study represents a sub-analysis of the OSGLA Registry and aimed to evaluate the short-term effects of a preservative-free glycerophosphoinositol-enriched hyaluronic acid formulation used as adjunctive therapy for GTOSD, compared with high-molecular-weight hyaluronic acid alone.

Both treatment regimens were associated with significant improvements in clinical signs and patient-reported outcomes after 1 month, with a broader intra-group pattern of improvement observed in patients receiving GPI/HA. However, these findings should be interpreted cautiously given the observational design and the limited magnitude of inter-group differences.

GTOSD is a well-recognized iatrogenic condition induced by long-term exposure to topical IOP-lowering medications and is characterized by epithelial damage, tear film instability, conjunctival hyperemia, immune activation, and neurosensory dysfunction. These alterations contribute to ocular discomfort, reduced quality of life, decreased adherence to therapy, and potentially worse surgical outcomes (17).

Within this framework, supportive therapies aimed at restoring tear film homeostasis and mitigating ocular surface inflammation are considered a key component of glaucoma management (5–7).

In the present cohort, adjunctive treatment with GPI/HA resulted in significant improvements in tear film stability, tear production, conjunctival hyperemia, epithelial integrity, and subjective symptom scores. BUT and STI values increased, indicating an improvement of tear film stability and volume, while BCH and CFS scores decreased, supporting a mitigation of the inflammation.

Overall, taken together, these changes were indicators of an overall improvement of ocular surface conditions. These objective findings were accompanied by significant reductions in SANDE and OSDI scores, supporting a consistent subjective perception of benefit. Importantly, effect size analysis revealed large effects for several parameters, including SANDE, OSDI, BCH, and CFS, and moderate effects for STI and BUT, suggesting that the observed changes were clinically meaningful and involved multiple dimensions of GTOSD.

Patients treated with HA alone also experienced significant improvements, particularly in conjunctival hyperemia, corneal staining, tear film stability, and symptom scores, confirming the established efficacy of preservative-free HMW-HA as a lubricant and epithelial-protective agent (18).

Importantly, background IOP-lowering therapy remained unchanged throughout follow-up, and the two groups showed comparable baseline treatment characteristics and preservative exposure. Therefore, the observed within-group improvements cannot be attributed to withdrawal of BAK-containing glaucoma medications or to changes in the background glaucoma regimen.

However, in this group, no significant improvement in tear production was observed, and the overall pattern of response appeared more confined to lubrication-related mechanisms.

Inter-group comparisons showed only modest differences at follow-up. Bulbar conjunctival hyperemia values were lower in the GPI/HA group, while NEI VFQ-25 scores were higher in the HA group. These findings should be interpreted cautiously, as BCH values were already significantly lower at baseline in patients receiving GPI/HA, and the study was not designed or powered to demonstrate comparative superiority. Nevertheless, the persistence of lower BCH values at follow-up may descriptively suggest a more favorable conjunctival profile in patients treated with GPI/HA, in line with the broader intra-group improvements observed in this cohort.

The potential additional effects observed with GPI/HA may be interpreted in light of the biological properties of GPI. GPI is an endogenous, water-soluble metabolite generated through phospholipase A_2_-dependent phosphoinositide metabolism and involved in the modulation of multiple cellular signaling processes (10). Experimental evidence suggests that GPI may participate in an endogenous negative-feedback mechanism that limits excessive inflammatory activation. In human monocytes exposed to inflammatory stimulation, exogenous GPI attenuated the activation of IKKα/β and MAPK signaling pathways, including p38, JNK, and ERK1/2, reduced NF-κB nuclear translocation and binding to inflammatory gene promoters, and ultimately decreased the expression and release of pro-inflammatory mediators (11). Beyond these anti-inflammatory effects, GPI-related metabolites have been implicated in the regulation of cell proliferation and survival and in cytoskeletal remodeling through modulation of Rho-family GTPases, suggesting broader effects on cellular homeostasis and adaptive responses to persistent stress (19). These biological properties may be relevant to GTOSD, in which chronic exposure to topical IOP-lowering therapy promotes persistent epithelial stress and dysregulated low-grade inflammation. In this context, the combination of GPI with HMW-HA may provide complementary actions: HA primarily contributes to lubrication, tear-film stabilization, viscoelastic support, and epithelial protection, whereas GPI may modulate cellular responses to persistent inflammatory and toxic stress. This biological rationale is also supported by previous clinical findings in patients with pollution-related ocular surface disease, in whom topical treatment with GPI in an HA vehicle was associated with improvements in ocular surface signs and symptoms (12). However, the mechanistic evidence supporting GPI activity is predominantly preclinical, and the present clinical findings cannot establish a specific molecular mechanism of action.

A relevant aspect of this study concerns the behavior of tear MMP-9. No statistically significant changes in MMP-9 immunoassay positivity were observed in either treatment group after 1 month. However, these findings should be interpreted cautiously. In the GPI/HA group, MMP-9 showed a large observed effect size despite not reaching statistical significance, suggesting that a potentially relevant treatment-associated change cannot be excluded. The relatively limited sample size may have reduced the ability to detect statistically significant differences for this biomarker. Larger prospective studies with adequately powered samples are required to confirm whether GPI/HA may influence inflammatory biomarker profiles in GTOSD (5).

In the HA group, MMP-9 positivity showed moderate to strong correlations with several clinical parameters, whereas in the GPI/HA group these associations were weak and largely non-significant. This finding should not be interpreted as evidence of absent biological activity, but rather reflects the complexity of inflammation in GTOSD. Unlike primary dry eye disease, GTOSD is characterized by chronic, low-grade inflammatory stress, and tear MMP-9 may represent a relatively insensitive biomarker in this context (3). The attenuation of correlations between MMP-9 positivity and both clinical signs and symptoms observed in the GPI/HA group may suggest a more homogeneous and stabilized ocular surface environment, in which clinical improvement is less directly linked to measurable biomarker expression. This interpretation remains exploratory and warrants confirmation in larger studies using quantitative inflammatory markers.

From a clinical perspective, both HA and GPI/HA appear to be valuable supports for managing GTOSD. The broader pattern of improvement observed in patients treated with GPI/HA, along with the large effect sizes detected across multiple parameters, suggests that formulations combining lubrication with non-pharmacological anti-inflammatory agents may offer some additional benefits. Improving ocular surface stability and comfort is clinically relevant not only for symptom relief and quality of life but also for maintaining long-term adherence to therapy and optimizing ocular surface conditions in view of potential future surgical interventions (6–8, 20–22). Consistently, previous real-world evidence from the OSGLA Registry showed that hyaluronic acid–based formulations enriched with natural anti-inflammatory agents, such as Aloe vera, were associated with short-term improvements in signs and symptoms of GTOSD, supporting the rationale for combining lubrication with non-pharmacological anti-inflammatory strategies (5).

Several limitations should be acknowledged. First, the observational and non-randomized design limits causal inference, and baseline imbalances, particularly in BCH, may have influenced inter-group comparisons. In addition, the 1-month follow-up was intended to capture short-term clinical changes and does not allow assessment of the durability of treatment effects. This relatively short observation period may also have been insufficient to detect changes in inflammatory biomarkers, particularly when assessed using a qualitative point-of-care assay.

Second, the sample size was relatively small, which limits the statistical power of the study and the robustness of between-group comparisons. Third, given the exploratory nature of the study and the absence of an *a priori* sample size calculation, treatment-associated changes were interpreted based on non-parametric effect size estimates rather than *post-hoc* power calculations. The limited sample size represents an important limitation, as it may have reduced the ability to detect statistically significant differences for some individual outcomes.

Fourth, tear MMP-9 was assessed using a qualitative point-of-care test, which may lack sensitivity for detecting subtle changes in chronic inflammatory states. As in other real-world studies, patient heterogeneity in terms of disease severity, duration of therapy, and treatment regimens may also have influenced outcomes. Further prospective studies with larger cohorts will be required to confirm these findings and clarify the role of MMP-9 as a biomarker of ocular surface changes associated with glaucoma therapy

Despite these limitations, the study has several strengths, including the use of a prospective multicenter registry with standardized data collection, the comprehensive assessment of both objective and patient-reported outcomes, and the evaluation of patients under stable background glaucoma therapy, thereby reducing potential treatment-related confounding.

In conclusion, this OSGLA Registry sub-analysis suggests that hyaluronic acid–based lubricants, with or without glycerophosphoinositol enrichment, are associated with short-term improvements in signs and symptoms of GTOSD. Although GPI/HA was associated with a broader pattern of intra-group improvements across objective parameters and patient-reported outcomes, these findings should be considered exploratory rather than definitive. Future randomized controlled studies with adequate sample size, longer follow-up, and quantitative biomarker assessment are warranted to better define the potential added value of GPI-enriched formulations in the management of GTOSD.

## Data Availability

The raw data supporting the conclusions of this article will be made available by the authors, without undue reservation.
